# Lipoteichoic Acid from *Staphylococcus aureus* Activates the Complement System via C3 Induction and CD55 Inhibition

**DOI:** 10.3390/microorganisms9061135

**Published:** 2021-05-24

**Authors:** Bong Jun Jung, Hangeun Kim, Kyoung Ok Jang, Seongjae Kim, Dae Kyun Chung

**Affiliations:** 1 Graduate School of Biotechnology, Kyung Hee University, Yongin 17104, Korea; bjjung@acebiome.com (B.J.J.); cko53@nate.com (K.O.J.); 2 Research and Development Center, Skin Biotechnology Center Co., Ltd., Yongin 17104, Korea; seongjaekim@khu.ac.kr; 3 Skin Biotechnology Center, Kyung Hee University, Suwon 16229, Korea

**Keywords:** lipoteichoic acid, complement, C3, CD55, membrane attack complex, IRAK-M

## Abstract

*Staphylococcus aureus* inhibits complement activity by secreting a variety of toxins. However, the underlying mechanism of complement component regulation by lipoteichoic acid (LTA), a cell wall component of *S. aureus*, has not been elucidated. In this study, we observed that aLTA (LTA of *S. aureus*) increased C3 expression in THP-1 cells. The mechanism of aLTA-mediated C3 induction includes an aLTA-toll-like receptor (TLR) 2 interaction, interleukin 1 receptor associated kinase (IRAK) 2 recruitment, and nuclear factor kappa B (NF-kB) activation. In HepG2 cells, C3 protein production begins to increase from 3 h and increases steadily until 48 h. On the other hand, CD55 levels increased up to 6 h after aLTA treatment and started to decrease after 24 h and levels were decreased at 48 h by more than 50% compared to untreated cells. The expression of CD55 in HepG2 cells was shown to be regulated by IRAK-M induced by aLTA. Serum C3 levels increased in mice injected with aLTA, which resulted in an increase in the amount and activity of the membrane attack complex (MAC). We also observed that CD55 mRNA was increased in the liver 24 h after aLTA injection, but was decreased 48 h after injection. These results suggest that aLTA increases complement levels via induction of C3 and inhibition of CD55, which may cause associated MAC-mediated liver damage.

## 1. Introduction

*Staphylococcus aureus* is a Gram-positive bacterium that is often found in the nose, respiratory system, and on the skin. While it can cause skin infections, respiratory infections, sinusitis, and food poisoning, it can also exist as normal flora. The emergence of methicillin-resistant *Staphylococcus aureus* (MRSA) and vancomycin-resistant *Staphylococcus aureus* (VRSA), which are resistant to antibiotics, have become an issue worldwide [1].

Complement, one of the first defense systems of the human body, is activated against *S. aureus* infection. The complement system is named as such since it complements the function of the immune system and phagocytosis and improves the ability of the host to attack the cell membranes of pathogens by promoting inflammation to remove pathogens. Complement is part of the innate immune system [2] that is activated by three routes including the classic, lectin, and alternative pathways and can detect and opsonize *S. aureus* to promote its phagocytosis by neutrophils in the blood and macrophages in tissues.

Factor C3 of the complement cascade plays a central role in the complement response and protection against *S. aureus* infection. Na et al. have shown that mice with C3 deficiency show susceptibility to *S. aureus* septic arthritis and display impaired host clearance, presumably due to reduced opsonization and phagocytosis of bacteria [3]. Conversely, *S. aureus* secretes several peptides to resist complement activity. Staphylococcal protein A (SpA) and *S. aureus* binder of immunoglobulin (Sbi) inhibit opsonophagocytic clearance of *S. aureus* by binding to the Fc region of IgG and complement factor C3 in serum [4,5]. Extracellular fibrinogen-binding protein (Efb) produced by *S. aureus* can bind to the alpha chain of C3 and inhibit both the classical and alternative pathways of complement activation [6].

Complement is activated by components of *S. aureus* such as crude cell walls (CCWs), purified cell walls (PCWs), peptidoglycan (PGN), and teichoic acid in normal serum [7]. Lipoteichoic acid (LTA) interacts with C1 and C1q, which inhibits complement activation capacity [8]. Kupffer cells, the tissue-resident macrophages in the liver, are able to capture circulating *S. aureus* through recognition of LTA by the complement receptor of immunoglobulin superfamily [9]. However, the mechanism of complement C3 expression regulation and complement activity by *S. aureus* LTA (aLTA) is not well known.

In the current study, we sought to elucidate the mechanism of C3 induction and CD55 inhibition in aLTA-treated THP-1 and HepG2 cells, respectively, and changes in complement activity by aLTA were observed in mice.

## 2. Materials and Methods

### 2.1. LTA Preparation

LTAs were purified from *S. aureus* (ATCC 25923; aLTA) and *L. plantarum* K8 (KCTC 10887BP; pLTA) as previously described [10]. Silver staining and endotoxin assays (GenScript, Piscataway, NJ, USA) were performed to test for contamination of protein and endotoxin, respectively. We confirmed that there was no protein contamination, and that the endotoxin contamination was less than 0.02 EU/mL in all LTA preparations.

### 2.2. Cell Culture

THP-1, a human monocytic cell line derived from an acute monocytic leukemia patient and HepG2, a human liver cancer cell line were cultured in RPMI 1640 and Dulbecco’s modified Eagle’s medium (DMEM), respectively, supplemented with 10% heat-inactivated fetal bovine serum (FBS) and 1% penicillin and streptomycin. The cells were incubated in a humidified 37 °C incubator with 5% CO_2_ atmosphere. For neutralization assays, anti-CD14 (mabg-hcd14), anti-TLR2 (pab-hstlr2), and anti-TLR4 (pab-hstlr4) neutralization antibodies (InvivoGen, San Diego, CA, USA) were pre-treated before the aLTA treatment in THP-1 cells.

### 2.3. Real-Time PCR

Cells were stimulated with pLTA and/or aLTA for the indicated time and total RNAs were extracted using RNA-Bee reagent (AMS Biotechnology, Cambridge, MA, USA). Total RNA (1.0 μg) was used for cDNA synthesis (iScript cDNA Synthesis kit; Bio-Rad, Hercules, CA, USA). The expression level of messenger RNA (mRNA) was measured by real-time PCR using the CFX Connect™ Real-Time PCR detection system (Bio-Rad), and the PCR products were detected with SYBR^Ⓡ^ Premix Ex II (TaKaRa, Japan). The sequences for the forward and reverse primer pairs are listed in Supplement Table S1. The comparative Δ−ΔCt method was carried out as outlined by Livak and Schmittgen [11]. Glyceraldehyde-3-phosphate dehydrogenase (GAPDH) was used to normalize the detected gene expression and fold change of experimental samples was estimated when untreated or control samples were set to 1.

### 2.4. Western Blot Analysis

THP-1 or HepG2 cells treated with aLTA were lysed with 2× reducing buffer and boiled for 5 min at 100 °C. Samples were loaded and resolved in 10% or 12% SDS-PAGE gels and proteins were transferred onto polyvinylidene fluoride (PVDF) membranes overnight at 40 V. The membranes were blocked with 5% bovine serum albumin (BSA) or skim milk in TBST (20 mM Tris-HCl, 150 mM NaCl, 0.05% Tween 20) for 1 h at room temperature (RT). After washing three times with TBST, membranes were incubated with anti-human C3, anti-human C5, anti-TLR2, anti-β-actin, anti-p65 (Santa Cruz Biotechnology, Inc., Dallas, TX, USA), anti- Interleukin 1 Receptor Associated Kinase (IRAK) 2, anti-IRAK-M, anti- suppressor of cytokine signaling (SOCS)-1 or anti-phospho p65 (Cell Signaling Technology Inc., Danvers, MA, USA) primary antibodies diluted in TBST (1:1000) for 2 h at RT and then washed three times with TBST. The membranes were incubated with secondary horseradish peroxidase (HRP)-conjugated anti-mouse or anti-rabbit antibody (1:2000 in TBST) for 2 h at RT. After washing three times with TBST, the membranes were treated with enhanced chemiluminescence (ECL) reagent and exposed to X-ray film. β-actin was used as the internal loading control.

### 2.5. Immunofluorescence

Cells were seeded in 6-well plates and treated with pLTA and aLTA for 0, 30, 60 or 120 min. After rinsing with phosphate-buffered saline (PBS) three times, cells were incubated with 4% formaldehyde in PBS for 15 min. Cells were incubated in methanol for 5 min in a freezer to penetrate the macrophage membranes and then rinsed with PBS for 5 min. Samples were blocked (10% BSA + 4% Triton X-100 in PBS) for 1 h, and 4% Triton X-100 was added to the blocking buffer to penetrate bacterial cell walls. Samples were reacted with anti-LTA antibody (diluted in 2% BSA + 4% Triton X-100) overnight at 4 °C. Subsequently, the samples were reacted with FITC-conjugated Alexa 488^®^ anti-mouse secondary antibodies for 2 h. A washing step (4% Triton X-100 in PBS) was applied between each step. The samples were mounted with Fluoro-Gel and photographed using fluorescence microscopy.

### 2.6. Bactericidal Assay

*Escherichia coli* DH5α was cultured in Luria-Bertani (LB) broth overnight. Then, the DH5α cells were washed and diluted with PBS. DH5α cells (1 × 10^4^) were cultured with mouse serum (1:50) at 37 °C for 1 h. The incubated DH5α cells were washed with PBS and spread on an LB agar plate. After overnight culturing, colony forming units (CFUs) were counted.

### 2.7. C9 Deposition Assay

The C9 deposition on DH5α cell surfaces was performed based on a previous study with minor modification [12]. Briefly, *E. coli* DH5α (1 × 10^7^) cells were incubated with aLTA-injected mouse serum or normal mouse serum (20% sera in a final volume of 100 μL) at 37 °C for 1 h. After a wash with PBS, opsonized log-phase bacteria were plated onto 96-well enzyme immunoassay (EIA) plates at 5 × 10^6^ cells per well and adhered by dry desiccation. Nonspecific binding sites were blocked with 200 μL 0.5% BSA in PBS and incubated for 30 min at 37 °C. After a wash, the plates were sequentially incubated at RT with 100 μL 1:100 (*v*/*v*) C9 antibody for 1 h, 100 μL 1:1000 HRP-conjugated secondary antibody for 30 min, and 100 μL substrate solution for visualizing HRP activity. The reaction was stopped with 50 μL 10% H_2_SO_4_, and the color reaction was detected at an optical density at 490 nm.

### 2.8. Mouse Study

Male BALB/c mice (6 weeks old) were purchased from Nara Bio (Gyeonggi, Korea). They were kept in individual cages at 24 ± 2 °C and 50 ± 10% moisture and fed nutritionally-balanced rodent food and sterilized water. The mice were cared for and used in accordance with the guidelines of the Animal Ethics Committee of Kyung Hee University (KHU14-021). Mice were injected with 50 mg/kg aLTA and blood samples were collected 24 h after injection for the analysis of serum complement components. Blood samples were left at RT for 30 min and then the sera were separated by centrifugation at 13,572× *g* for 20 min. The sera were used for membrane attack complex (MAC) and C3 quantity, C9 deposition assays, and bacterial killing assays. The expression levels of serum C3 and MAC were examined by commercially available C3 ELISA kit (ab157711, Abcam, Cambridge, MA, USA) and MAC ELISA kit (MBS-261074, MyBioSource, San Diego, CA, USA) according to the manufacturer’s introduction.

In the alternative study, mice were repeatedly injected with 50 mg/kg aLTA from 1 to 4 times (on the first, second, fourth, and seventh day) and sera and organs were collected 24 h after injection.

### 2.9. Statistical Analysis

All the experiments were repeated at least three times. The data shown are representative results of the means ± SD of triplicate experiments. Statistical analyses were conducted using unpaired two-tailed *t*-tests, one-way ANOVA followed by Tukey’s honestly significant difference (HSD) post-hoc tests or two-way ANOVA. Prism 5 software was used for the analysis (GraphPad software Inc., San Diego, CA, USA). A *p* < 0.05 was considered significant.

## 3. Results

### 3.1. C3 Expression Is Significantly Increased by the aLTA Treatment

**Figure 1:**
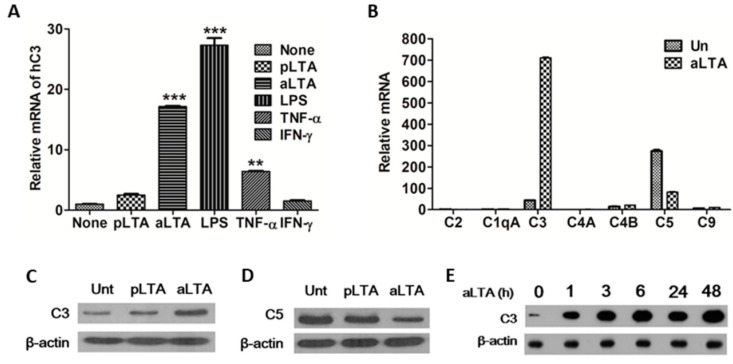
C3 induction by aLTA (lipoteichoic acid isolated from *S. aureus* cell wall). (**A**) THP-1 cells were stimulated with the indicated ligands for 6 h and mRNA levels of C3 were measured using real-time PCR. (**B**) The mRNA expression level of complement components induced by aLTA was compared to C2 (C2 was set to 1-fold). C3 (**C**) and C5 (**D**) protein levels were examined by Western blot after THP-1 cells were stimulated with pLTA or aLTA for 24 h. (**E**) THP-1 cells were stimulated with aLTA for the indicated times and C3 protein was examined by Western blot. The amount of ligand used in the experiment was 100 μg/mL aLTA and pLTA (LTA isolated from *L. plantarum*), 500 ng/mL lipopolysaccharide (LPS), and 10 ng/mL tumor necrosis factor alpha (TNF-α) and interferon gamma (IFN-γ). The mRNA level was normalized with glyceraldehyde 3-phosphate dehydrogenase (GAPDH). The data are displayed as the mean ± SD of three independent experiments. Statistical analysis was conducted with a one-way ANOVA. ** *p* < 0.01, *** *p* < 0.001 as compared to the control (none). β-actin was used as the internal loading control.

We examined the C3 mRNA expression level by real-time PCR. When THP-1 cells were treated with aLTA for 6 h, C3 mRNA was increased by 18-fold as compared to the untreated cells. On the other hand, pLTA (lipoteichoic acid isolated from *Lactobacillus plantarum* cell wall) treatment did not have a significant effect on C3 mRNA expression. LPS, a cell wall component of Gram-negative bacteria, was more inducible than aLTA. Among the cytokines, TNF-α increased C3 mRNA, whereas IFN-γ did not (Figure 1A). The mRNA expression of complement components including C1qA, C2, C4A, C4B, C5, and C9 were varied depending on the inducer used for THP-1 stimulation (Supplementary Figure S1). In particular, the expression of most complement components was increased by aLTA, whereas C5 showed a tendency to decrease significantly (Supplementary Figure S1E). When C2 mRNA was fixed at 1 and the expression levels of other complement components were compared, C3 mRNA expression was 43-fold higher than that of C2 and was increased to 711-fold when cells were treated with aLTA. Compared to C2, C5 was 273-fold higher, while the aLTA treatment decreased C5 by 82-fold compared to C2 (Figure 1B). Protein levels of C3 and C5 also coincided with mRNA levels. The aLTA increased C3 protein level (Figure 1C), whereas C5 protein was decreased in aLTA-treated cells (Figure 1D). Since complement C3 plays a central role in complement activation, we focused on the regulation of C3 by aLTA in the current study. When THP-1 cells were treated with 100 μg/mL aLTA for the indicated times, C3 protein was dramatically increased from 1 to 48 h (Figure 1E).

### 3.2. Toll-Like Recptor (TLR) 2 Is Involved in aLTA-Mediated C3 Expression

**Figure 2:**
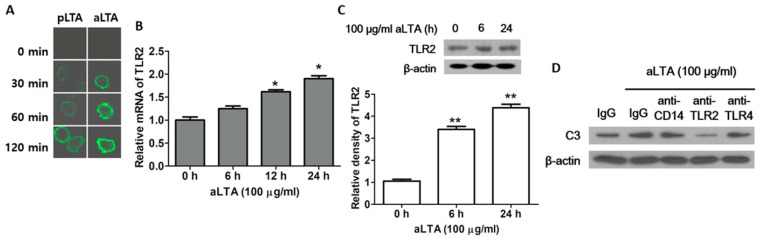
Toll-Like Receptor (TLR) 2-mediated C3 induction in aLTA-treated THP-1 cells. (**A**) Cell surface attachment of LTA was visualized by immunofluorescence staining after 10 μg/mL pLTA- or aLTA-treated THP-1 cells were stained with anti-LTA antibody. (**B**) TLR2 mRNA level was measured using real-time PCR. (**C**) TLR2 protein level was examined by Western blot (upper panel) and the relative density was generated (lower panel). (**D**) THP-1 cells were pretreated with 10 μg/mL neutralization antibodies for 30 min and retreated with 100 μg/mL aLTA. C3 protein level was examined by Western blot. The mRNA level was normalized with GAPDH. β-actin was used as the internal loading control. The data are displayed as the mean ± SD of three independent experiments. Statistical analysis was conducted with a one-way ANOVA. * *p* < 0.05, ** *p* < 0.01 as compared to 0 h.

To examine the interaction between LTA and TLR2, immunofluorescence assays were performed after treatment with pLTA and aLTA in THP-1 cells. As shown in Figure 2A, the cell surface attachment of aLTA seemed stronger than that of pLTA. This result may explain why aLTA increases C3 expression more than pLTA. Next, we examined the mRNA and protein levels of TLR2 after the aLTA treatment. TLR2 mRNA was increased in a time-dependent manner (Figure 2B), whereas the protein was significantly increased from 6 h (Figure 2C). When cells were blocked with neutralization antibodies, such as anti-CD14, anti-TLR2 or anti-TLR4 antibody, C3 expression was blocked by TLR2, but not by CD14 and TLR4, suggesting that TLR2 plays an important role in aLTA-mediated C3 expression in THP-1 cells (Figure 2D).

### 3.3. IRAK2 Plays an Important Role in aLTA-TLR2-Mediated C3 Expression

**Figure 3:**
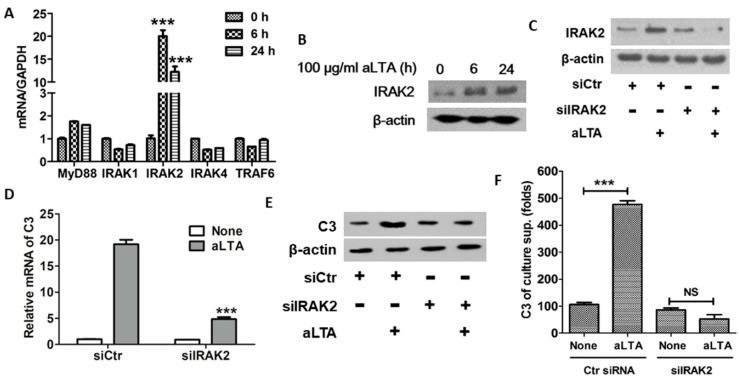
IRAK2 is involved in aLTA-TLR2-mediated signaling. (**A**) The mRNA level of adaptive molecules was measured using real-time PCR after aLTA treatment for the indicated times. (**B**) IRAK2 protein level was examined by Western blot after aLTA treatment for the indicated times. (**C**–**F**) THP-1 cells were transiently transfected with IRAK2 siRNA and then stimulated with 100 μg/mL aLTA for 6 h. IRAK2 protein level was examined by Western blot (**C**). C3 mRNA level was examined by real-time PCR (**D**). C3 protein level was examined by Western blot (**E**). Secreted C3 level was examined by ELISA with culture supernatants (**F**). The mRNA level was normalized with GAPDH. β-actin was used as the internal loading control. The data are displayed as the mean ± SD of three independent experiments. Statistical analysis was conducted with an unpaired two-tailed *t*-test. *** *p* < 0.001 as compared to 0 h or siCtr.

To examine the role of adaptive proteins in aLTA-TLR2-mediated signaling pathways, adaptor mRNA levels were examined. When THP-1 cells were treated with aLTA, IRAK2 mRNA was significantly increased at 6 h treatment, and then decreased at 24 h. Other factors such as MyD88, IRAK1, IRAK4, and TRAF6 were not altered in the mRNA level by aLTA (Figure 3A). IRAK-2 protein was increased from 6 h and was greatly increased at 24 h of stimulation (Figure 3B). When IRAK2 expression was suppressed in IRAK2 siRNA-transfected cells (Figure 3C), aLTA-mediated C3 mRNA and protein expression were significantly diminished as compared to cells transfected with control siRNA. The C3 mRNA and protein expression was decreased in IRAK2 knock-down cells, while control siRNA-transfected cells increased C3 mRNA expression when transfected cells were stimulated with aLTA (Figure 3D,E). The secreted C3 level was also increased in control siRNA-infected cells but was not increased in IRAK2 knock-down cells (Figure 3F). These data suggest that IRAK2 is involved in aLTA-TLR2-mediated C3 expression.

### 3.4. NF-κB Is a Central Mediator for aLTA-Mediated C3 Production

**Figure 4:**
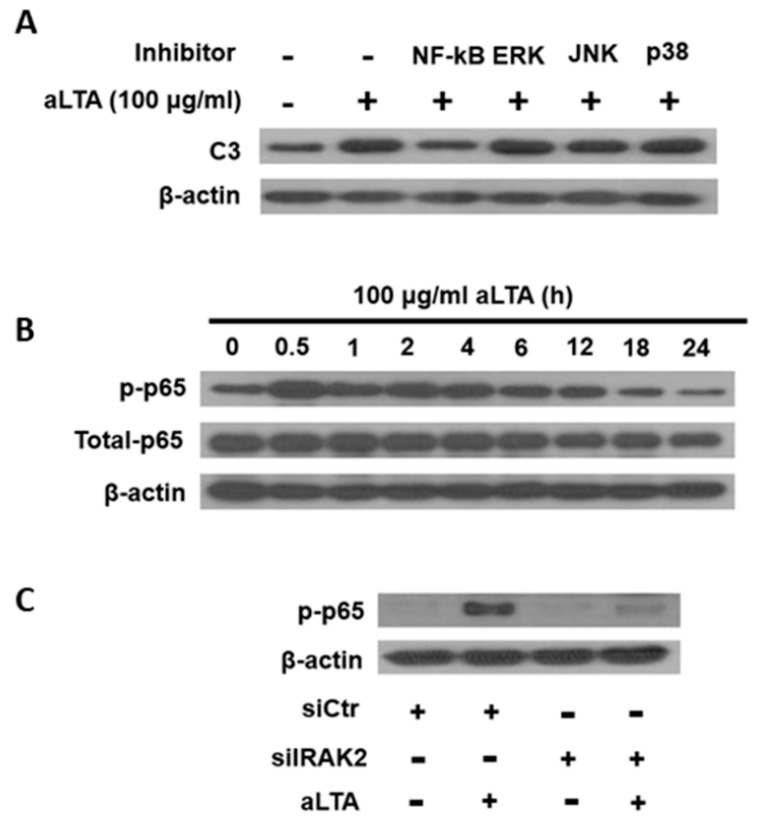
NF-κB signaling is involved in aLTA-mediated C3 production. (**A**) THP-1 cells were pretreated with 10 µM inhibitors for signaling molecules and then treated with 100 μg/mL aLTA for 24 h. C3 protein was examined by Western blot. (**B**) The total and phosphorylation of NF-κB p65 subunit were examined by Western blot after THP-1 cells were treated with 100 μg/mL aLTA for the indicated times. (**C**) THP-1 cells were transiently transfected with IRAK2 siRNA and then stimulated with 100 μg/mL aLTA for 1 h. The level of phosphorylated p65 was examined by Western blot. β-actin was used as the internal loading control. Inhibitors used in this experiment were NF-κB activation inhibitor (#481406), ERK inhibitor (#328006), JNK inhibitor II (#420119), and SB203580 (#559389) purchased from Sigma-Aldrich.

THP-1 cells were pretreated with NF-κB, ERK, JNK, and p38 inhibitors and then retreated with 100 μg/mL aLTA for 24 h. C3 protein expression was inhibited by NF-κB inhibitor, but not by the other inhibitors, indicating that NF-κB plays a major role in the aLTA-mediated signaling pathway (Figure 4A). Phosphorylation of the NF-κB p65 subunit was increased from 0.5 h and returned to a steady state 18 h after aLTA stimulation (Figure 4B). When cells were transiently transfected with IRAK2 siRNA, the phosphorylation of p65 was not induced by aLTA, suggesting that IRAK2 is a major component for the activation of NF-κB (Figure 4C).

### 3.5. The aLTA Down-Regulated CD55 Production in HepG2 Cells through the Induction of IRAK-M

**Figure 5:**
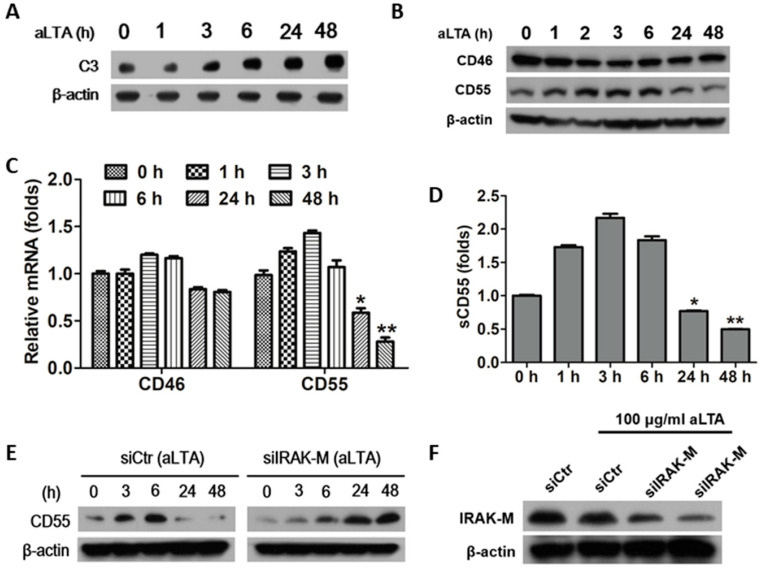
CD55 is decreased in aLTA-treated HepG2 cells. HepG2 cells were treated with 10 μg/mL aLTA for the indicated times and C3 level was examined by Western blot (**A**), CD46 and CD55 level was examined by Western blot (**B**), mRNA level of CD46 and CD55 was measured using real-time PCR (**C**), and soluble CD55 level was examined by indirect ELISA from culture supernatants (**D**). (**E**,**F**) HepG2 cells were transiently transfected with IRAK-M siRNA and control siRNA and then stimulated with 100 μg/mL aLTA for the indicated times. CD55 (**E**) and IRAK-M (**F**) protein levels were examined by Western blot. The mRNA level was normalized with GAPDH. Statistical analysis was conducted with a one-way ANOVA. * *p* < 0.05, ** *p* < 0.01 as compared to 0 h. β-actin was used as the internal loading control.

Since the liver is a major source of complement, we confirmed the expression of membrane cofactor protein (MCP, CD46) and decay-accelerating factor (DAF, CD55), which regulate the activity of C3, in aLTA-treated HepG2 cells. Similar in THP-1 cells, HepG2 cells continuously increased C3 production up to 48 h in response to aLTA (Figure 5A). However, membrane-bound complement regulatory proteins (mCRPs) showed different expression patterns from C3. CD46 decreased between 2 and 24 h after aLTA treatment. On the other hand, CD55 increased between 2 and 6 h and decreased significantly after 24 h (Figure 5B). This inhibition pattern of CD55 appears specifically at the mRNA level, which was significantly reduced after 24 h compared to the untreated cells (Figure 5C). The amount of CD55 secreted into the culture medium also increased compared to the control between 1 and 6 h, but significantly decreased compared to the control after 24 h (Figure 5D). These results indicate that CD55 increased at the initial stage of aLTA treatment, which resulted in the inhibition of C3 cleavage and complement activation, but prolonged exposure induced excessive complement activation by decreasing CD55. IRAK-M negatively regulates TLR signaling by preventing the dissociation of IRAK1 and IRAK4 from MyD88 [13]. When HepG2 cells were treated with aLTA, the expression of IRAK-M peaked at 6 h, and then decreased at 24 and 48 h. The transient transfection of siRNA for IRAK-M showed that CD55 increased in a time-dependent manner, indicating that IRAK-M negatively regulates CD55 expression (Figure 5E).

### 3.6. The aLTA Treatment Increased C3 Activation in Mice

**Figure 6:**
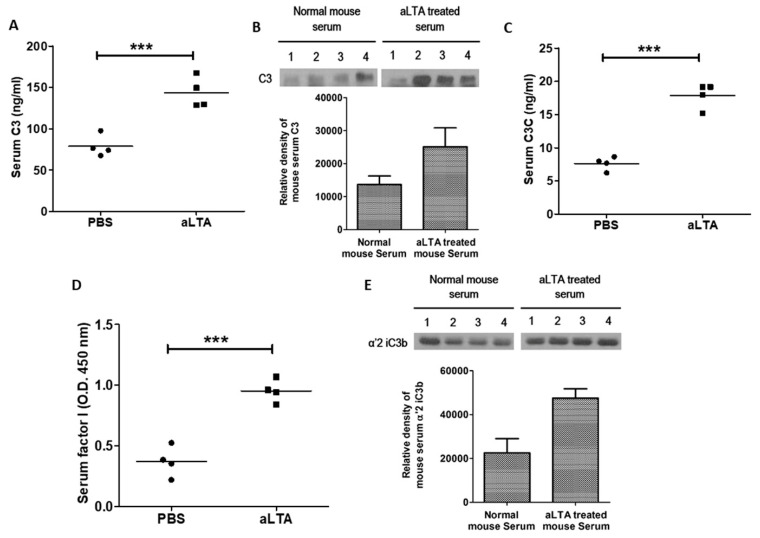
The aLTA increases C3 activity in mouse sera. Mice were injected with 50 mg/kg aLTA for 24 h and then blood was collected (*n* = 4). (**A**) The serum C3 level was examined by ELISA. (**B**) C3 protein level of mouse sera was examined by Western blot (upper panel) and the densitometry is shown (lower panel). (**C**) The serum C3C level was examined by ELISA. (**D**) The serum factor I level was examined by indirect ELISA. (**E**) The iC3b level of mouse sera was examined by Western blot (upper panel) and the densitometry is shown (lower panel). Statistical analysis was conducted with an unpaired two-tailed *t*-test. *** *p* < 0.001.

The aLTA treatment increased serum C3 level, which was confirmed by ELISA (Figure 6A) and Western blot analysis (Figure 6B), respectively. C3 convertase was also increased by the aLTA treatment, suggesting that aLTA increases complement activation in mice (Figure 6C). In addition, we observed increased serum factor I (Figure 6D), which increased C3b cleavage and produced iC3b (Figure 6E). Complement factor I, also known as C3b/C4b inactivator, cleaves the C3b fragment at two sites to form iC3b [14]. The iC3b secreted by apoptotic tumor cells prevents complete maturation of dendritic cells and induces antigen-specific silencing or resistance [15]. Thus, our data suggest that aLTA can inactivate immune cells by increasing iC3b.

### 3.7. Activation of MAC by aLTA Induces Liver Damage

**Figure 7:**
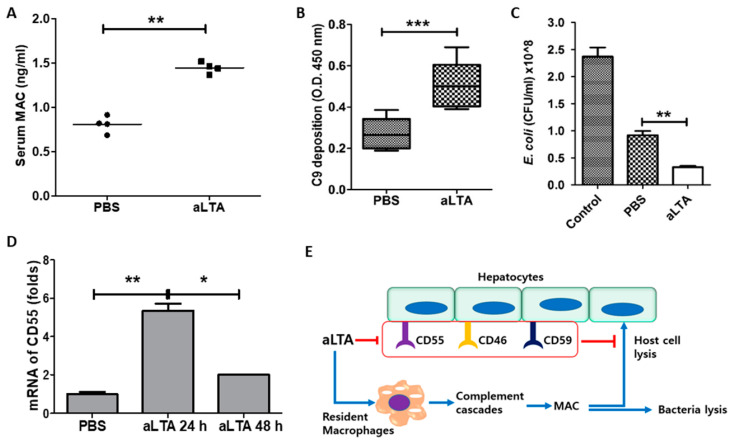
The aLTA increases membrane attack complex (MAC) activity in mice. Mice were injected with 50 mg/kg aLTA for 24 h and then blood was collected (*n* = 4). (**A**) Serum MAC level was examined by ELISA. (**B**) C9 deposition on the bacterial cell surface was examined as described in the Materials and Methods. (**C**) Bactericidal activity was examined with mouse sera as described in the Materials and Methods. Statistical analysis was conducted with an unpaired two-tailed *t*-test. ** *p* < 0.01, *** *p* < 0.001. (**D**) CD55 mRNA level was examined by real-time PCR with mouse liver biopsies. Statistical analysis was conducted with a one-way ANOVA. * *p* < 0.05; ** *p* < 0.01. (**E**) Schematic diagram for the putative complement regulating mechanism of aLTA.

The induction of C3 and C3C by aLTA increased the formation of MAC. We observed an increased serum MAC level in aLTA-treated mice (Figure 7A). Activation of the complement system by aLTA was also confirmed by the increase of C9 deposition on the bacterial cell surface (Figure 7B), and aLTA-mediated C9 deposition increased the bactericidal effects (Figure 7C). Overactivation of the complement system induces organ damage [16], and abnormal regulation of the complement system can be controlled by CD55. Thus, we examined the level of CD55 from mouse liver. As shown in Figure 7D, aLTA significantly increased CD55 mRNA 24 h post-injection, but was decreased after 48 h. While C3 production was continuously increased by aLTA, the inhibition of CD55 on the cell surface may cause organ damage, as well as the bacterial killing via MAC formed by the aLTA-mediated signaling pathway and complement cascades (Figure 7E).

### 3.8. Repeated Treatment with aLTA Induces Organ Injury as Well as MAC Activation

**Figure 8:**
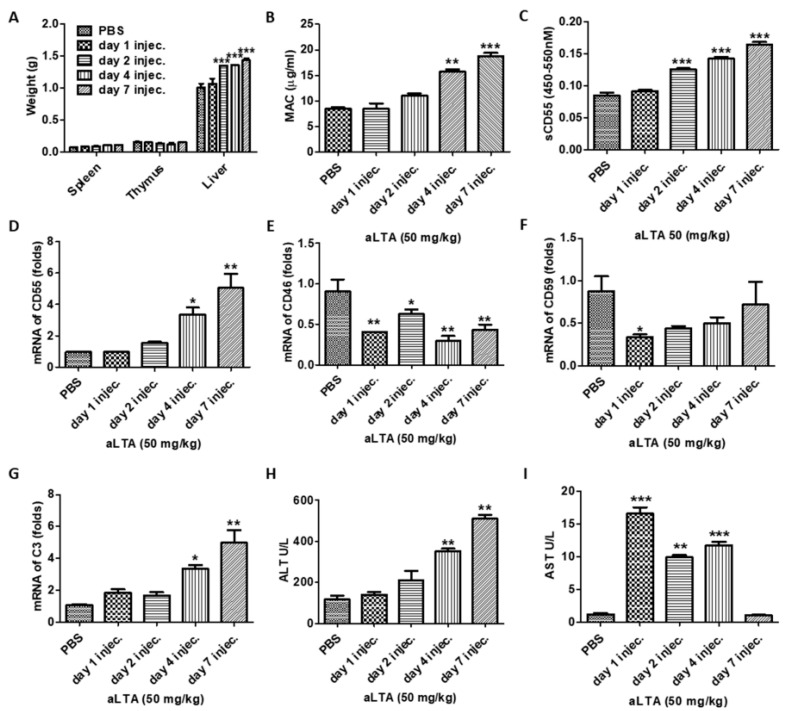
The aLTA induces organ failure. Mice (*n* = 4) were repeatedly injected with 50 mg/kg aLTA on the first, second, fourth, and seventh days. (**A**) The weight of the spleen, thymus, and liver was examined. (**B**) The amount of MAC was examined by ELISA from the aLTA-injected mouse serum. (**C**) Secreted CD55 in the sera was examined by indirect ELISA. (**D**–**G**) The mRNA level of CD55 (**D**), CD46 (**E**), CD59 (**F**), and C3 (**G**) from liver was measured using real-time PCR. Serum alanine aminotransferase (ALT) (**H**) and aspartate aminotransferase (AST) (**I**) levels were examined by ELISA. Statistical analysis was conducted with a one-way ANOVA. * *p* < 0.05; ** *p* < 0.01; *** *p* < 0.001.

To investigate the long-term effects of aLTA, we repeatedly injected 50 mg/kg aLTA into mice. Serum and the organs were collected after the first, second, fourth, and seventh day of injection, respectively. In the analysis, the weight of the spleen slightly increased significantly, but the weight of the liver was increased significantly. On the other hand, the thymus weight did not change significantly (Figure 8A). The final product of the complement cascade, MAC was increased as aLTA was repeatedly injected (Figure 8B). Unexpectedly, the secreted CD55 (Figure 8C) and mRNA level of CD55 (Figure 8D) were increased in the blood and liver, respectively. Instead, mRNA of CD46 and CD59 in the liver were decreased by repeated injection of aLTA (Figure 8E,F). In addition, the C3 mRNA level was increased by aLTA (Figure 8G), suggesting that the C3-mediated complement can be activated, and the liver can be damaged by MAC attack. In fact, it was confirmed that the level of alanine aminotransferase (ALT) and aspartate aminotransferase (AST), which indicates the degree of liver damage, increases with the repeated injection of aLTA (Figure 8H,I).

## 4. Discussion

The complement system seems to be closely related to liver damage. The inhibition of either C3 or C5 in the *Escherichia coli* sepsis mouse model attenuated liver injury by reducing ALT and AST levels [17]. ALT is an enzyme found in most liver and kidney cells, and in small amounts in the heart and muscles. In healthy people, the level of ALT in the blood is low, but when the liver is damaged, ALT is released into the bloodstream, usually before clear symptoms of liver damage, such as jaundice, develop [18]. Due to this phenomenon, ALT is used as a useful test to detect liver damage. On the other hand, AST is an enzyme present in the heart, kidney, brain, and muscle as well as liver cells, and its concentration increases when these cells are damaged [19]. In this study, ALT levels increased when aLTA was repeatedly injected, with a concomitant increase of MAC, showing that MAC and ALT have a close relationship. As a separate experiment, we observed that both ALT and MAC levels increased after repeatedly injecting 1 × 10^7^ live *S. aureus* into mice. Similar to aLTA, *S. aureus* injection induced an increase in the volume and weight of both the liver and spleen, indicating that the cell wall component LTA may also be involved in the induction of sepsis due to *S. aureus* infection (Supplement Figure S2).

In the early stages of aLTA stimulation, the immune system seems to activate complement via increasing the C3 production to treat invading bacteria. Tissues slightly increase CD55 expression on their cell surface to inactivate complement overactivated by pathogens. In our study, CD55 expression is significantly decreased under 24 h stimulation in the in vitro model. According to our findings, the decrease in CD55 seems to be regulated by IRAK-M, which was increased by aLTA stimulation. The decrease in inhibitors capable of controlling C3 results in increased complement activity and increased tissue damage by MAC. The expression pattern of CD46 was opposite to that of CD55, and this trend appears differently in vivo as CD55 levels increased by repeated injection of aLTA, whereas CD46 tended to decrease. CD59, another complement regulatory protein, showed a similar expression pattern to CD55 in HaCaT cells, but it was inhibited in aLTA-injected mice. CD59 inhibits the activity of MAC, thus, a decrease in CD59 can cause tissue damage by MAC [20]. In fact, blood levels of C3, C3C, and MAC were significantly increased by aLTA, suggesting that the inhibitory system for complement may not be activated in aLTA-injected mice. However, these results are not consistent with our previous study using *S. aureus*-injected mice. When mice were received a single I.P. injection with 1 × 10^8^ CFU *S. aureus*, the levels of C3, C3C, and MAC decreased in the serum. The bactericidal activity of sera was also lower in *S. aureus*-injected mice as compared to normal mice.

The complement system is activated to eradicate foreign pathogens. On the other hand, pathogens have evolved to escape the attack of the complement system. For example, *S. aureus* increases the production of CD55 in host cells, which inactivates the cleavage of C3. Unlike *S. aureus*, aLTA, a purified cell wall component of *S. aureus*, activated the complement system in the mouse model. This result is probably due to the use of highly concentrated aLTA. As we show in the results, aLTA steadily increased the expression of C3, which increased the activation of MAC. On the other hand, when *S. aureus* was injected, it would not have sufficient LTA to induce C3 expression. When *S. aureus* enters the body, it inhibits the activity of the complement system, and when toxins such as *S. aureus* LTA are injected, tissue damage due to excessive complement activity may occur.

The negative regulation of TLR signaling is a very important cellular event to maintain homeostasis of the immune system [21]. In the current study, we found that aLTA increased IRAK-M expression, which inhibits TLR signaling by preventing the dissociation of IRAK1/4 or IRAK2/4 from MyD88 [13]. IRAK-M levels increased from 3 h of aLTA treatment and showed a maximum level at 6 h. On the other hand, the phosphorylation of NF-kB peaked at 0.5 h and then showed a tendency to decrease. The increase in NF-kB activity by aLTA probably induced the expression of C3 and IRAK-M. Increased IRAK-M could have selectively inhibited CD55, which can be hypothesized since the expression of CD55 was suppressed, whereas the expression of C3 continued to increase until 48 h. The difference in C3 and CD55 expression by aLTA or IRAK-M may be related to the mRNA stability of these genes, but further studies are needed to support this hypothesis.

Interestingly, some LTAs isolated from *E. hirae* and *S. pyogenes* did not stimulate strong TLR2 responses. Instead, both *E. hirae* LTA and *S. pyogenes* LTA mounted a reasonable response in a TLR4 reporter gene assay [22]. Biochemical studies using cell wall component-deficient mutant bacteria clearly demonstrated that bacterial lipoproteins, but not LTA or PGN, act as native TLR2 ligands [23,24,25]. This is supported by the analyses of TLR2 ectodomain crystal structures in complex with a lipopeptide [26,27], and by studies using *lgt S. aureus* mutants [23,24,25,28,29], and using a LTA-deficient *S. aureus ltaS* (LTA synthase) mutant [23,24,25]. Therefore, some researchers have suggested that only the lipoproteins are the true TLR2 ligands, and others may contain lipoproteins as contaminants during their preparations. On the other hand, many researchers seem to believe that LTA acts as a TLR2 agonist. For example, aLTA inhibits LPS-induced B cell proliferation in vitro, ex vivo, and in vivo models, but the effect was not observed in the splenocytes from TLR2-deficient mice, suggesting that TLR2 is involved in aLTA-mediated signaling [30]. Two independent studies by Wang and Jiang used sodium phenylbutyrate and matrine, respectively, to inhibit the aLTA-induced TLR2 signaling pathway [31,32]. Whether LTA is an agonist of TLR2 still seems to be controversial, but many researchers still recognize LTA as a TLR2 agonist. In the present study, aLTA was considered as an agonist of TLR2.

The LTA of *S. aureus* is one of the most potent virulence factors and increases the expression of complement C3 as well as a strong inflammatory response. On the other hand, CD55, which is a complement regulator, is inhibited by aLTA, which can cause damage to organs such as the liver by inducing abnormal complement activity. This study revealed the role of aLTA in the regulation of complement activity and the induction of organ damage by the MAC.

## Data Availability

The data presented in this study are available on request from the corresponding authors.

## Supplementary Materials

The following are available online at https://www.mdpi.com/article/10.3390/microorganisms9061135/s1, Figure S1: The variation of mRNA level of complement components in THP-1 cells stimulated by ligands, Figure S2: The *S. aureus* induced organ failure, Table S1: Primer set used in the study.
