# Supplementary material for: Lipoteichoic Acid from Staphylococcus aureus Activates the Complement System via C3 Induction and CD55 Inhibition
Source: Microorganisms. 2021 May 24;9(6):1135. doi: 10.3390/microorganisms9061135 (PMC8225101; doi:10.3390/microorganisms9061135)
Supplement: Supplementary file 1 [file microorganisms-09-01135-s001.zip › Supplement Table 1.pdf]

Supplement Table 1. Primer set used in the study.

Gene	Primer	Sequence (5'→3')
C1qA	Forward	AACATCAAGGACCAGCCGAG
	Reverse	ACCGTGTCGAAGATGACCAC
C2	Forward	CACTGTGCATCAGGGAGACCT
	Reverse	CCCCATGACCAAAGCGGAA
C3	Forward	ACCAGCAGACCGTAACCATC
	Reverse	GCAGCCTTGACTTCCACTTC
C4A	Forward	CCGTCTGTCTGTCTGCTACC
	Reverse	CTTTCACTACCTGTCCTCGGG
C4B	Forward	CAAGACAGAGCAGTGGAGCA
	Reverse	ACAACCAAGCCGCATAGGAA
C5	Forward	TGTCGTCGCAAGCCAGCTCC
	Reverse	TGCCAATGCCTTGAATTTCC
C9	Forward	ATTGTGAAAGTGAGCCCCGT
	Reverse	ATCCCATAGCCTGCTGTTCG
CD46	Forward	GTGAGGAGCCACCAACATTT
	Reverse	GCGGTCATCTGAGACAGGT

CD55	Forward	CAGCACCACCACAAATTGAC
	Reverse	CTGAACTGTTGGTGGGACCT
CD59	Forward	CCGCTTGAGGGAAAATGAG
	Reverse	CAGAAATGGAGTCACCAGCA
TLR2	Forward	TCCTGCTAAGAGACTCCTCTGT
	Reverse	TGGGGAGTGCCCCAAATACT
MyD88	Forward	TCGACTGAAGTTGTGTGTGT
	Reverse	AATCATCAGAGACAACCACC
IRAK1	Forward	ATCTACAAGAAGCACCTGGA
	Reverse	TCTCTAGCCTCTCGTACACC
IRAK2	Forward	GGAGATCATCCACAGCAACG
	Reverse	GCCTGTACCCTCAGAAAGCC
IRAK4	Forward	AATGATGCTGATTCCACTTC
	Reverse	GCTGGTGAACCTTCTTAATG
IRAK-M	Forward	TTGGTCCTGGGCACAGAAAA
	Reverse	TCGAATGTGCCAAGGGAGTG
TRAF6	Forward	AAGAGAACACCCAGTCACAC
	Reverse	GTCTTGTCTTACAAGGCGAC

GAPDH	Forward	AAGGTCGGAGTCAACGGATT
	Reverse	GCAGTGAGGGTCTCTCTCCT
