# Supplementary figures and images for: Lipoteichoic Acid from Staphylococcus aureus Activates the Complement System via C3 Induction and CD55 Inhibition
Source: Microorganisms. 2021 May 24;9(6):1135. doi: 10.3390/microorganisms9061135 (PMC8225101; doi:10.3390/microorganisms9061135)

## Slide 1
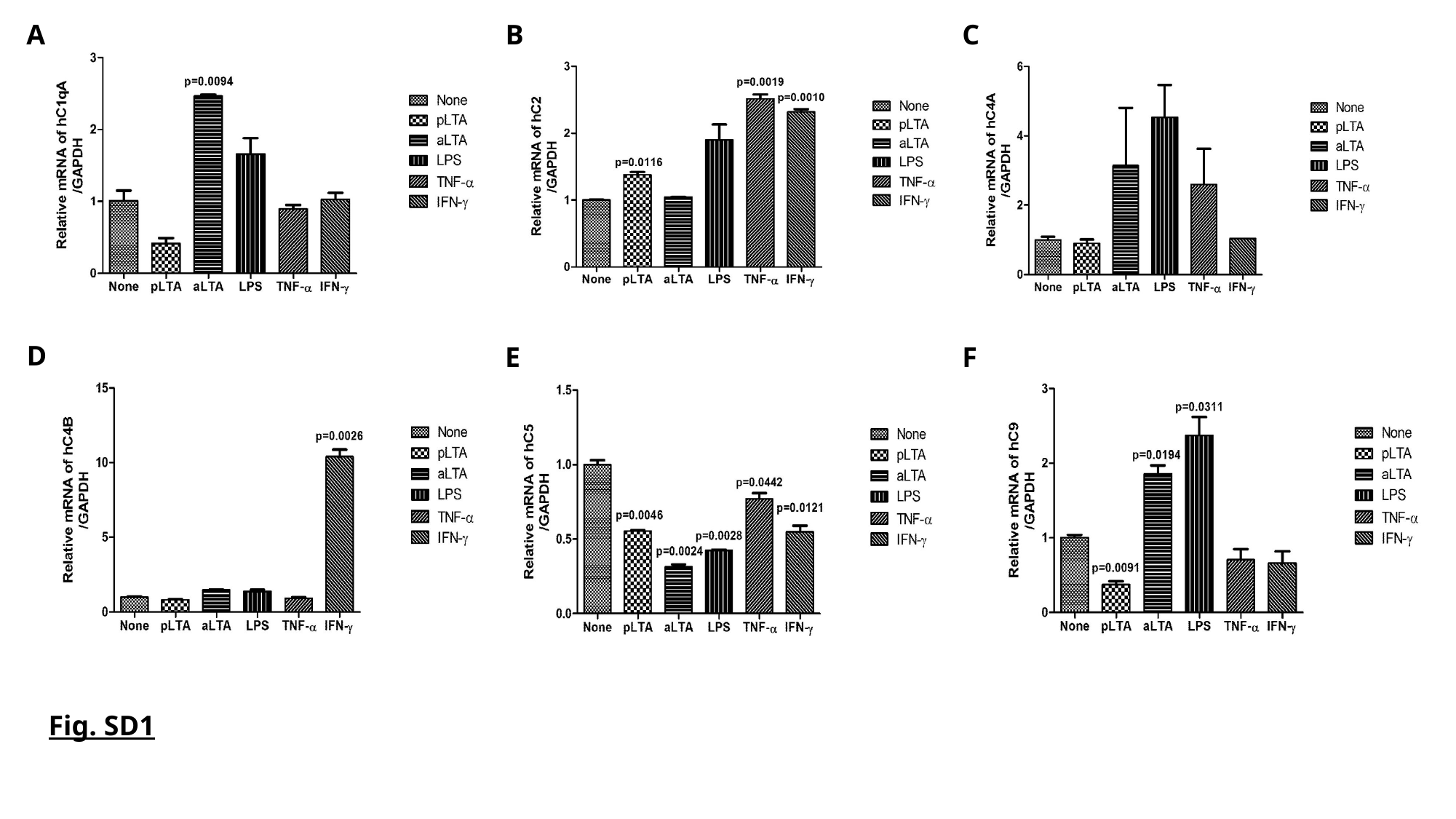

A
B
C
D
E
F
Fig. SD1

## Slide 2
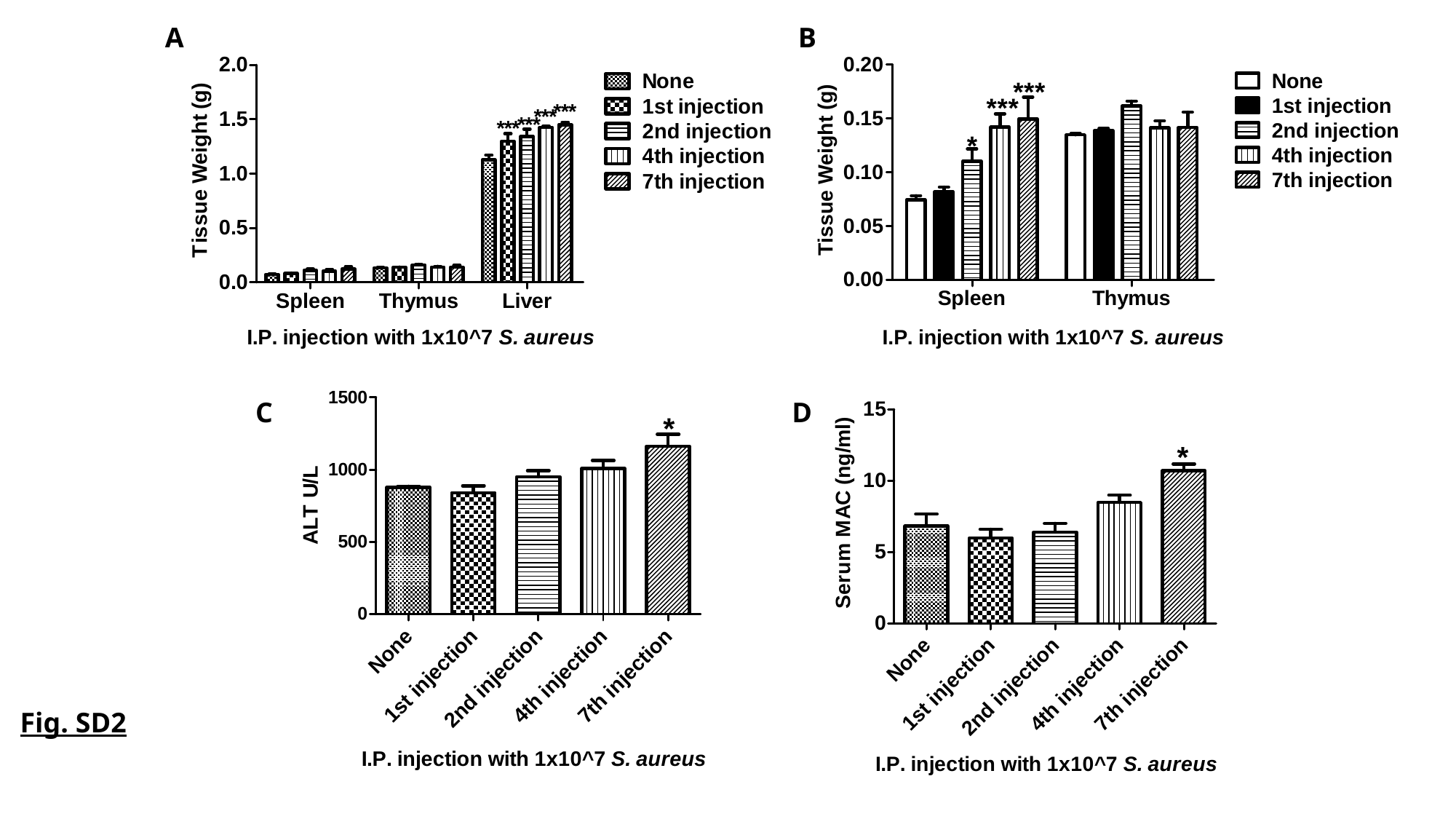

A
B
C
D
Fig. SD2

Supplement: Supplementary file 1 [file microorganisms-09-01135-s001.zip › Supplement Figure.pptx]
